# Determination of Δ^9^-tetrahydrocannabinol, 11-nor-carboxy-Δ^9^-tetrahydrocannabinol and cannabidiol in human plasma and urine after a commercial cannabidiol oil product intake

**DOI:** 10.1007/s11419-024-00686-0

**Published:** 2024-04-09

**Authors:** Ioannis Papoutsis, Vasiliki Hatzidouka, Stamatina-Panagoula Ntoupa, Apostolis Angelis, Artemisia Dona, Emmanouil Sakelliadis, Chara Spiliopoulou

**Affiliations:** 1 Department of Forensic Medicine and Toxicology, Medical School, National and Kapodistrian University of Athens, Athens, Greece; 2 Division of Pharmaceutical Chemistry, Faculty of Pharmacy, National and Kapodistrian University of Athens, Athens, Greece; 3 Department of Pharmacognosy and Natural Products Chemistry, Faculty of Pharmacy, National and Kapodistrian University of Athens, Athens, Greece

**Keywords:** CBD products, Δ^9^-tetrahydrocannabinol, 11-nor-carboxy-Δ^9^-tetrahydrocannabinol, Plasma, Urine, Forensic investigation

## Abstract

**Purpose:**

Cannabidiol (CBD) products are widely used for pain relief, sleep improvement, management of seizures etc. Although the concentrations of Δ^9^-tetrahydrocannabinol (Δ^9^-THC) in these products are low (≤0.3% w/w), it is important to investigate if its presence and/or that of its metabolite 11-nor-carboxy-Δ^9^-THC, is traceable in plasma and urine samples of individuals who take CBD oil products.

**Methods:**

A sensitive GC/MS method for the determination of Δ^9^-THC, 11-nor-carboxy-Δ^9^-THC and CBD in plasma and urine samples was developed and validated. The sample preparation procedure included protein precipitation for plasma samples and hydrolysis for urine samples, solid-phase extraction and finally derivatization with *N*,O-bis(trimethylsilyl)trifluoroacetamide) with 1% trimethylchlorosilane.

**Results:**

For all analytes, the LOD and LOQ were 0.06 and 0.20 ng/mL, respectively. The calibration curves were linear (*R*^2^ ≥ 0.992), and absolute recoveries were ≥91.7%. Accuracy and precision were within the accepted range. From the analysis of biologic samples of 10 human participants who were taking CBD oil, it was realized that Δ^9^-THC was not detected in urine, while 11-nor-carboxy-Δ^9^-THC (0.69–23.06 ng/mL) and CBD (0.29–96.78 ng/mL) were found in all urine samples. Regarding plasma samples, Δ^9^-THC (0.21–0.62 ng/mL) was detected in 10, 11-nor-carboxy-Δ^9^-THC (0.20–2.44 ng/mL) in 35, while CBD (0.20–1.58 ng/mL) in 25 out of 38 samples, respectively.

**Conclusion:**

The results showed that Δ^9^-THC is likely to be found in plasma although at low concentrations. In addition, the detection of 11-nor-carboxy-Δ^9^-THC in both urine and plasma samples raises questions and concerns for the proper interpretation of toxicological results, especially considering Greece’s zero tolerance law applied in DUID and workplace cases.

## Introduction

*Cannabis sativa* (*C. sativa*) has been one of the most extensively used recreational drugs over the years [1]. Its use in traditional medicine dates older than 4000 years; however just in recent years, therapeutic applications of cannabis products have shown an upward trend worldwide [2]. The main cannabinoids contained in *C. sativa* are Δ^9^-tetrahydrocannabinol (Δ^9^-THC) and cannabidiol (CBD), which are also the major pharmacologically active compounds of the plant [3].

Δ^9^-THC presents strong psychoactive properties and induces the “high” effect that occurs after smoking; however, it also has serious side effects, limiting its use as a medicinal drug [4]. CBD, a non-psychoactive cannabinoid, has gained widespread attention the last decade, due to its high antioxidant and anti-inflammatory properties [5], and its potential use as anticonvulsant, anxiolytic, neuroprotective, and antibiotic agent [6, 7].

Recreational use of cannabis is performed most likely by smoking or vaporization, while medicinal use of cannabis follows orally administration or vaporization [8, 9]. Cannabis-derived products for medicinal use have been approved by the US Food and Drug Administration (FDA) and European Medicines Agency (EMA). Sativex (nabiximols) is an oromucosal spray that contains Δ^9^-THC and CBD in equal parts and is used for Multiple Sclerosis spasticity. Epidiolex is an oral solution of CBD and is prescribed for treatment of pediatric seizures associated with two rare forms of epilepsy [10, 11].

At the same time, non-medical products for human use of cannabinoids, especially CBD, are also available in the market, as dietary supplements, liquids for electronic cigarettes, cosmetics, or even for veterinary use [12]. In some European countries, galenic CBD oils, tinctures, capsules and crystal are also prescribed and prepared to alleviate chronic pain, pediatric epilepsy, multiple sclerosis or other pathologic conditions [13]. These products are mainly derived from hemp varieties that are high in CBD and low in Δ^9^-THC, they lack official approval for medical use and no prescription is required for their purchase [14].

Cannabidiol is not included in the European Union List of Controlled Substances [15]; thus, many companies are allowed to produce and distribute products with CBD, while thorough quality controls are optional and there are several concerns for the content and purity of products that are sold. In the USA, CBD belongs to Schedule 1 of the Controlled Substances Act, while in 2018 Drug Enforcement Administration rescheduled Epidiolex as a Schedule 5 substance [13].

Although a regulatory framework for CBD products is not well defined yet, there is a strict legislation regarding the content of Δ^9^-THC in these preparations, varying among different countries. In Europe, the USA and Canada, cannabis varieties with Δ^9^-THC content of less than 0.2% (Europe) and 0.3% (USA and Canada) are allowed to be cultivated. In some varieties of cannabis, known as “CBD-rich”, the CBD content is greater than 12%, while the Δ^9^-THC content is usually greater than 0.3%. Consequently, depending on the hemp variety that is used for CBD extraction, Δ^9^-THC levels in final products may vary, and even may exceed the law limit [16].

The illegal use of cannabis as well as cannabis-derived products for medical purposes, have led to the development of several methods for the identification of cannabinoids in biologic materials for clinical and forensic purposes. The main analytes of interest are Δ^9^-THC and its metabolites, while there are also a great number of methods that also include CBD to the analytes of interest. Numerous methods for the determination of Δ^9^-THC and its metabolites, 11-hydroxy-Δ^9^-tetrahydrocannabinol (11-OH-Δ^9^-THC) and 11-nor-carboxy-Δ^9^-THC and/or CBD as have been published in the scientific literature, in several biologic materials, such as whole blood [17–24], plasma [25–36], serum [34], urine [7, 17, 30, 35, 37–39] or oral fluid [7, 17, 40–42]. These methods are based on gas chromatography (GC) [25, 26, 31, 32, 34, 36, 40, 43–46] or liquid chromatography (LC) [17, 19, 20, 22–24, 28, 39, 41, 47–51], mainly coupled to mass spectrometry (MS) [17, 19, 20, 22–26, 28, 31, 32, 34, 36, 39–41, 43–51].

The interpretation of a toxicological result in the case of DUID, workplace drug testing or post-mortem investigation is of great importance. The expanding use of CBD products has raised concerns regarding the interpretation of toxicological results of individuals taking legal CBD products [52].

Despite the trace levels of Δ^9^-THC in CBD products, it is questionable whether Δ^9^-THC as well as its major metabolite 11-nor-carboxy-Δ^9^-THC can be detected in biologic materials, after consumption of these products, so misinterpretation of the legal intake of CBD products with the recreational use of cannabis is possible. In Greece, several CBD products are available containing different concentrations of CBD ranging from 1 to 15% w/v. The aim of this study was to investigate if low Δ^9^-THC levels contained in CBD products could give a positive result on cannabis testing. For this purpose, the development and validation of an analytical method for the determination of CBD, Δ^9^-THC and 11-nor-carboxy-Δ^9^-THC in biologic materials was crucial, to quantitate and evaluate CBD, Δ^9^-THC and 11-nor-carboxy-Δ^9^-THC levels obtained in plasma and urine samples of individuals after repeated intake of a CBD product.

## Materials and methods

### Chemicals and reagents

CBD, Δ^9^-THC, 11-nor-carboxy-Δ^9^-THC, Δ^9^-THC-d3 and 11-nor-carboxy-Δ^9^-THC-d3 were purchased from LGC Promochem (Molsheim, France). All solvents (methanol, acetonitrile, acetone, glacial acetic acid, *n-*hexane, and ethyl acetate) were HPLC grade and were purchased from Merck (Darmstadt, Germany). The derivatization reagent *N, O-*bis(trimethylsilyl)-trifluoracetamide (BSTFA) with 1% trimethylchlorosilane (TMCS) was provided from Fluka (Steinheim, Germany). Sodium acetate was purchased from Sigma-Aldrich (Steinheim, Germany). For solid-phase extraction (SPE), Bond Elut LRC Certify II (sorbent mass 200 mg, column volume 10 mL) columns were obtained from Agilent Technologies (Lake Forest, CA, USA). The enzyme β-glucuronidase from Helix pomatia was provided by Merck (Darmstadt, Germany).

Human plasma and urine were collected from healthy donors. The absence of CBD, Δ^9^-THC and 11-nor-carboxy-Δ^9^-THC or other drugs was confirmed, and then the samples were pooled before spiking for the preparation of calibration and quality control (QC) samples.

Participants were between ages of 27 and 60 years old. Ten healthy volunteers, 5 males and 5 females, were given written details of the study and written informed consent was obtained. The used CBD formulations were available in retail that was labeled as hemp extract of 2.8% (w/v) CBD.

### Calibrators and quality control samples

Standard stock solution of CBD, Δ^9^-THC and 11-nor-carboxy-Δ^9^-THC in acetonitrile were at a concentration of 1.0 mg/mL. By proper dilutions with acetonitrile, working solutions of the analytes were prepared at concentrations of 0.004, 0.010, 0.020, 0.060, 0.20, 0.40, 1.00 μg/mL for the calibrators and 0.012, 0.300, 0.800 μg/mL for the QC samples. An aliquot of 50 μL of the corresponding working solution was spiked in 1.0 mL of plasma or urine for the preparation of calibration and QC samples at the final concentrations of 0.20, 0.50, 1.00, 3.00, 10.00, 20.00, 50.00 ng/mL and 0.60, 15.00, 40.00 ng/mL (low, medium and high QC concentration), respectively. For I.S, a working solution contained Δ^9^-THC-d3 and 11-nor-carboxy-Δ^9^-THC-d3 was prepared at a concentration of 0.300 μg/mL.

### GC/MS analysis and apparatus

A GC/MSD (model 6890N/5975) instrumentation, supplied by Agilent Technologies (IL, USA) coupled with a DB-5MS fused silica column (30 m × 0.25 mm i.d., film thickness 0.25 μm) was used for the chromatographic analysis. Helium was used as the carrier gas at a flow rate of 1.0 mL/min. A 1 μL aliquot was injected in the splitless mode using an Agilent 7683B Series auto-sampler system. The optimized GC conditions were as follows: The initial column temperature was 100 °C which was held for 1 min and then increased to 300 °C at a rate of 30 °C/min, where it was held for 5 min. Injector, ion source and interface temperatures were set at 280, 230 and 280 °C, respectively. Electron impact ionization was used, combined with selected ion monitoring (SIM) mode. The mass fragments used for the identification of the silylated analytes were as follows: *m/z* **390**, 337 and 301 for CBD, *m/z* **371**, 386, 303 for Δ^9^-THC, *m/z* **371**, 473 and 488 for 11-nor-carboxy-Δ^9^-THC *m/z* **374** for both internal standards Δ^9^-THC-d3 and 11-nor-carboxy-Δ^9^-THC-d3, whereas the bold marked ions were used for the quantification of analytes.

An MT 19 vortex (Chiltern, London, UK) was used for the mixing of standards and samples during their preparation. A 691 digital pH-meter (Metrohm, Herisau, Switzerland) with a glass electrode was used for pH adjustments. An evaporating unit connected with nitrogen (Reacti-Vap PIERCE Model 18,780, Rockford, IL, USA) was used for the evaporation of all samples. Centrifugation was performed with a Sigma 4K10 centrifuge (Osterade, Germany).

### Sample preparation

A two-step extraction procedure was performed, as described below, with two elutions, one for the neutral analytes, and one for the acidic analytes. 50 μL of I.S. working solution was added to 1.0 mL of calibration, QC and unknown samples (15.0 ng/mL). For the protein precipitation of plasma samples, 2.0 mL of acetonitrile was added to all samples under vortex-mixing. After the centrifugation of the samples at 3000 rpm for 5 min, the organic supernatant phase was transferred into a clean glass tube, and was evaporated under a gentle stream of N_2_ at 40 °C to approximately 0.5 mL. The pH of the samples was then adjusted to 7.0 with the addition of 5.0 mL of sodium acetate buffer (pH 7.0): methanol (95:5, v/v) and then SPE was carried out with Bond Elut Certify II LRC columns. The columns were conditioned with 2 mL of methanol and 2 mL of sodium acetate buffer (pH 7.0): methanol (95:5, v/v) prior to sample loading. After sample application, the columns were washed with 2 mL sodium acetate buffer (pH 7.0): methanol (95:5, v/v), dried under high vacuum (>10 mm Hg) for 10 s and 100 μL of acetone was added. After drying the columns under high vacuum (>10 mm Hg) for 5 min, CBD, and THC were eluted with the addition of 2 mL of a mixture of hexane: ethyl acetate (90:10 v/v) twice. The columns were washed again with 3 mL of methanol: distilled H_2_O (50:50 v/v), high vacuum for 10 s was applied, and then 100 μL of ethyl acetate was added. High vacuum for 5 min was applied and 11-nor-carboxy- Δ^9^-THC was eluted with the addition of 2 mL of a mixture of hexane: ethyl acetate: glacial acetic acid (90/10/1, v/v/v) twice. The eluates were evaporated to dryness under the stream of N_2_ and the residues were derivatized by adding 50 μL acetonitrile and 50 μL of BSTFA with 1% TMCS at 70 °C for 30 min. The derivatives were transferred to GC–MS vials and 1 μL was injected into the system.

Regarding urine samples, an additional step of glucuronide cleavage was performed. For alkaline hydrolysis of 11-nor-carboxy-Δ^9^-THC glucuronide, to 1.0 mL urine, 200 μL 10 M KOH was added to each sample and the samples were incubated at 50 °C for 15 min. The samples were then cooled, and the pH was adjusted to 4.5 by adding HCl 0.1 M solution dropwise. In addition, for enzyme hydrolysis of Δ^9^-THC and CBD glucuronides, 3000 IU of b-glucuronidase H. pomatia were added to each sample and incubated at 37 °C for 16 h. The samples were then cooled at room temperature and the pH was adjusted to 7 by adding 5 mL sodium acetate buffer (pH 7.0): methanol (95:5, v/v) and the compounds were extracted as described above.

### Study design

The selected CBD formulation was analyzed before administration, to determine the Δ^9^-THC concentration and any variations of the indicated CBD concentration. A quantity of the formulation was diluted to methanol. A methanolic calibration curve was constructed and used for the quantification of CBD and Δ^9^-THC. The concentrations of CBD and Δ^9^-THC were calculated 3.33% (w/w) and 0.14% (w/w), respectively.

Ten healthy volunteers were given a written explanation and written informed consent was obtained before joining the study. The participants were receiving 6 drops (200.4 ± 7.8 mg) of the specific formulation once daily for five days, through sublingual administration. The CBD and Δ^9^-THC daily dose was 6.61 ± 0.26 mg and 0.28 ± 0.01 mg, respectively. To our knowledge, the chosen formulation had one of the lowest labeled concentrations of CBD in the local market. The selected dose is a relatively low dose that an individual can receive, and it was selected to evaluate how the intake of lower doses of Δ^9^-THC contained in the total cannabis extract, affects the drug testing, since in higher concentrations of relative formulations, this effect is expected to be more intense.

Blood samples (~5 mL) were collected during the 5th day of administration, at four different time points, before the administration and 1, 2 and 3 h after the administration of the CBD formulation. Blood collection tubes containing K_2_ EDTA were used, and after centrifugation at 3000 rpm for 10 min, plasma samples were obtained. Participants provided also two urine samples, the morning urine, and 3 h after receiving the CBD product. All samples were analyzed at the day of their collection.

## Results

### Method validation

Following the international guidelines [53, 54], the developed method was validated and several parameters, such as selectivity, specificity, sensitivity, linearity, absolute recovery, accuracy and precision were evaluated.

Selectivity and specificity were assessed to test any endogenous or exogenous interference. Selectivity was evaluated by the chromatograms obtained from the analysis of six different blank plasma and urine samples to identify if any endogenous compound interferes at the retention time of the analytes and internal standards and no matrix effect was observed from the blank samples analyzed. Specificity was determined after analyzing spiked samples with a wide range of commonly used drugs and their metabolites (morphine, codeine, 6-acetyl-morphine, cocaine, benzoylecgonine, ECME, methadone, buprenorphine, nor-buprenorphine, fentanyl, nor-fentanyl, pethidine, tramadol, amphetamine, methamphetamine, MDMA, MDA, MDEA, MBDB, ephedrine, ketamine, nor-ketamine, alprazolam, bromazepam, 7-amino-flunitrazepam, diazepam, nordiazepam, lorazepam, amisulpride, biperiden, clomipramine, risperidone, hydroxy-risperidone, levomepromazine, olanzapine, quetiapine, zolpidem, amitriptyline, citalopram, mirtazapine, sertraline, desmethyl-sertraline, venlafaxine, nor-venlafaxine, and paracetamol) (*n* = 45), at a final concentration of 500 ng/mL. There were no observed-exogenous interferences with the analytes of interest.

Method sensitivity was evaluated by the analysis of both spiked plasma and urine samples prepared at different concentrations for the determination of the limit of detection (LOD) and quantification (LOQ) for each analyte. LOD and LOQ were determined as the concentration resulting in a peak area with a signal-to-noise ≥3 and ≥10, respectively, that allows quantification of the analytes with acceptable accuracy and precision (≤20%). LOD and LOQ were found to be 0.06 and 0.20 ng/mL, respectively, for each analyte.

Linearity was determined through the construction of a seven-point calibration curve for each analyte of interest and biologic material in four different days. The calibration curve ranged from 0.20 to 50.0 ng/mL for CBD, Δ^9^-THC and 11-nor-carboxy-Δ^9^-THC. Graphs were constructed correlating the concentration of each analyte with the peak area ratio of the analyte to that of the respective internal standard. The regression line for each compound was calculated using the method of least squares with a weighting factor of 1/*x*^2^ (*x* was the concentration of each analyte), and was found to be >0.992 for all analytes. The % relative standard deviation (RSD) of slopes was also calculated and was found less than 4.6, 2.5 and 2.3% for CBD, Δ^9^-THC and 11-nor-carboxy-Δ^9^-THC, respectively.

Absolute recovery of each analyte was assessed at three QC concentration levels, by calculating the ratio of the response of the analyte after extraction of a spiked plasma or urine sample to the response of a reference standard solution multiplied by 100. Six replicates of each sample were analyzed, and absolute recovery for all QC concentration levels ranged between 95.4 and 102.4% for CBD, between 92.0 and 106.6% for Δ^9^-THC, and between 91.7 and 96.5% for 11-nor-carboxy-Δ^9^-THC.

Accuracy and precision were assessed by the analysis of spiked plasma and urine samples of each analyte at three QC concentration levels. Precision was expressed as the coefficients of variation (% RSD) and accepted RSD values were lower than 15% for all QC concentrations. Accuracy was calculated as the percentage difference of the determined concentration from the theoretical concentration (% *E*_r_) and accepted % *E*_r_ values were within 15% for all QC concentrations. Intraday variation was determined by running six samples of each QC concentration of each analyte on the same day, while interday precision and accuracy was assessed by a total of 24 samples at each QC concentration in four different days. Detailed results for the three analytes of interest are shown in Tables 1 and 2 for plasma and urine, respectively.

**Table 1:** Intraday and interday accuracies and precisions of the developed method for the determination of CBD, Δ^9^-THC and 11-nor-carboxy-Δ^9^-THC in plasma at three QC concentrations

Analyte	QC concentration (ng/mL)	Intraday (*n* = 6)		Interday (*n* = 24)	
		Accuracy (% *E*_r_)	Precision (% RSD)	Accuracy (% *E*_r_)	Precision (% RSD)
CBD	0.60	4.17	6.67	− 8.33	6.67
	15.0	− 6.14	5.18	− 9.33	4.84
	40.0	− 5.04	4.92	− 11.90	4.66
Δ^9^-THC	0.60	1.95	4.92	− 5.00	3.28
	15.0	− 4.58	5.97	− 5.80	4.15
	40.0	− 5.00	4.45	− 9.65	4.74
11-nor-carboxy-Δ^9^-THC	0.60	− 3.06	8.47	− 8.33	6.67
	15.0	− 2.56	5.95	− 8.60	4.46
	40.0	− 4.05	8.38	− 6.72	5.22

**Table 2:** Intraday and interday accuracies and precisions of the developed method for the determination of CBD, Δ^9^-THC and 11-nor-carboxy-Δ^9^-THC in urine at three QC concentrations

Analyte	QC concentration (ng/mL)	Intraday (*n* = 6)		Interday (*n* = 24)	
		Accuracy (% *E*_r_)	Precision (% RSD)	Accuracy (% *E*_r_)	Precision (% RSD)
CBD	0.60	0.77	2.45	− 0.19	3.31
	15.0	− 0.30	5.30	− 1.05	3.54
	40.0	− 4.10	3.02	− 4.91	2.74
Δ^9^-THC	0.60	− 5.62	5.37	− 4.02	2.76
	15.0	− 2.17	1.77	− 1.53	1.13
	40.0	− 4.32	1.64	− 4.19	1.74
11-nor-carboxy-Δ^9^-THC	0.60	− 2.51	2.17	− 2.86	3.15
	15.0	0.02	0.89	0.26	3.41
	40.0	− 1.99	1.69	− 2.61	2.84

### Determination of analytes in plasma and urine samples

After validation, the developed method was applied to plasma and urine samples of individuals that were following a controlled once daily administration of a CBD formulation. The CBD and Δ^9^-THC daily dose was 6.61 ± 0.26 mg and 0.28 ± 0.01 mg, respectively. Blood samples were collected during the 5th day before administration and after 1, 2, and 3 h after administration, while two urine samples were also collected, morning urine (before administration) and 3 h after administration. Plasma and urine samples were analyzed, and CBD, Δ^9^-THC and 11-nor-carboxy-Δ^9^-THC were quantified using the previously described method. The concentrations of the substances in plasma and urine samples are presented in Table 3.

**Table 3:** The concentrations of Δ^9^-THC, 11-nor-carboxy-Δ^9^-THC, and CBD in plasma and urine samples of participants at different sampling times

Participant	Analyte	Plasma concentration (ng/mL) Before administration	Plasma concentration (ng/mL) 1 h post administration	Plasma concentration (ng/mL) 2 h post administration	Plasma concentration (ng/mL) 3 h post administration	First morning urine concentration (ng/mL)	Urine concentration (ng/mL) 3 h post administration
1	CBD	n.d	n.d	n.d	0.87	0.29	4.46
	THC	n.d	n.d	n.d	n.d	n.d	n.d
	11-nor-carboxy-Δ^9^-THC	0.26	0.26	0.52	1.81	1.48	3.92
2	CBD	n.d	<LOQ	0.30	<LOQ	1.06	76.36
	THC	n.d	n.d	n.d	n.d	n.d	n.d
	11-nor-carboxy-Δ^9^-THC	0.58	2.26	2.42	1.97	1.99	5.09
3	CBD	n.d	<LOQ	0.63	0.52	2.45	4.64
	THC	n.d	n.d	<LOQ	<LOQ	n.d	n.d
	11-nor-carboxy-Δ^9^-THC	0.20	0.28	1.93	1.28	1.77	0.69
4	CBD	n.d	1.58	0.42	0.28	2.52	14.10
	THC	n.d	0.45	0.21	n.d	n.d	n.d
	11-nor-carboxy-Δ^9^-THC	n.d	1.79	n.d	n.d	3.11	1.74
5	CBD	n.d	0.76	0.34	n.d	11.62	61.92
	THC	n.d	0.31	n.d	n.d	n.d	n.d
	11-nor-carboxy-Δ^9^-THC	0.60	1.24	2.44	2.27	9.85	23.06
6	CBD	n.d	0.49	1.09	0.33	3.19	96.78
	THC	n.d	0.22	0.28	n.d	n.d	n.d
	11-nor-carboxy-Δ^9^-THC	n.d	0.53	0.96	0.42	5.90	4.90
7	CBD	n.d	0.68	1.00	0.27	1.92	18.12
	THC	n.d	<LOQ	<LOQ	*n.d*	*n.d*	*n.d*
	11-nor-carboxy-Δ^9^-THC	<LOQ	0.70	1.17	0.78	5.82	3.36
8	CBD	n.d	0.53	0.31	<LOQ	1.60	1.88
	THC	n.d	0.62	n.d	n.d	n.d	n.d
	11-nor-carboxy-Δ^9^-THC	<LOQ	<LOQ	0.58	0.44	1.40	0.75
9	CBD	n.d	0.20	0.40	–	10.55	96.59
	THC	n.d	n.d	n.d	–	n.d	n.d
	11-nor-carboxy-Δ^9^-THC	<LOQ	0.61	1.03	–	7.25	4.57
10	CBD	n.d	<LOQ	<LOQ	–	9.23	20.01
	THC	n.d	n.d	n.d	–	n.d	n.d
	11-nor-carboxy-Δ^9^-THC	0.23	0.24	0.24	–	1.90	3.90

## Discussion

From the results of this study, it was observed that Δ^9^-THC was not detectable in any of the urine samples, while it was mostly found at low concentrations, in plasma samples of the participants collected 1 and 2 h after administration. These concentrations ranged between 0.21 and 0.62 ng/mL, while four samples had concentrations below LOQ. Δ^9^-THC was not detected in any plasma samples collected prior to administration, indicating no accumulation and rapid biotransformation to metabolites. This is also supported by the fact that only one participant had detectable levels of Δ^9^-THC, below LOQ, 3 h after administration. Consequently, it seems that the detection of Δ^9^-THC in plasma indicates the recent administration of the formulation.

11-nor-carboxy-Δ^9^-THC, the inactive metabolite of Δ^9^-THC, was detected in the majority of participants’ plasma samples and in all urine samples. Urine concentrations for 11-nor-carboxy-Δ^9^-THC ranged between 0.69 and 23.06 ng/mL while those in plasma ranged between 0.2 and 2.44 ng/mL. Four plasma samples had concentrations below LOQ. Mean *C*_max_ in plasma samples was 1.54 ng/mL and was achieved 2 h after administration, showing a later peak compared to the parent compound Δ^9^-THC. The results of our study suggest that 11-nor-carboxy-Δ^9^-THC accumulates in the body, since it was detected both in plasma samples collected prior to the last administration, as well as in morning urine, indicating that 11-nor-carboxy-Δ^9^-THC is detectable in both plasma and urine for at least 24 h.

CBD was found in the most plasma samples collected, at low concentrations ranged from 0.2 to 1.58 ng/mL, while six samples presented concentrations below LOQ. Higher concentrations in plasma were observed between 1 and 2 h after administration, with a mean *C*_max_ of 0.70 ng/mL. In urine samples, CBD was detected in all collected samples, at concentrations between 0.29 and 96.78 ng/mL. In all cases, urine samples collected 3 h after administration showed higher concentrations compared to morning urine, indicating that CBD follows rapid elimination and that the amount of detected CBD is due to the last administration of the formulation. The detection of CBD in morning urine of all participants argues with its accumulation in the body, while its absence in plasma samples collected prior to administration, implies that CBD was present at concentrations lower than LOD, despite the administration during the previous days.

In the scientific literature there are several published studies concerning the determination of cannabinoids in biologic samples after the administration of legal cannabis-derived products. In most studies, CBD-rich cannabis is administered in individuals by vaporization [7, 42, 55–57], however oral uptake of pure CBD [56, 57], CBD-rich extract [58, 59], oil solution or sublingual products [60–63] has also been studied. Cannabinoids have also been determined after the administration of the FDA-approved Epidiolex [64] and Sativex [60, 65]. These studies are mainly focusing on the pharmacokinetic profile of cannabinoids, evaluating parameters that may affect their bioavailability, as well as their effect on vital signs. Furthermore, in some recent studies healthy adult dogs have received veterinary hemp products containing CBD, at doses of 4 mg/kg/day [66] or 5 and 10 mg/kg/day [67].

Comparison with previously published studies may not be valid, since different doses and routes of administration have been used. In some studies of a CBD oil solution, the administered doses to healthy volunteers contained 50 mg [60] or 100 mg [63] of CBD, doses much higher to the one administered in our study (6.61 ± 0.26 mg) [60], while there are also other published studies with low doses of CBD using cannabis decoction or oil products [61, 62].

Our study showed that Δ^9^-THC is not accumulated in plasma, since no sample collected prior to administration had detectable Δ^9^-THC. This observation is consistent with a previous study, where no accumulation was observed after smoking of CBD-rich cannabis [68]. Due to rapid metabolism, the detectable window for Δ^9^-THC in plasma is short, and Δ^9^-THC was only detected for 1 and 2 h after administration. Earlier study has supported that Δ^9^-THC can be detected in blood samples obtained until 40 min, but not after 1 and 2 h after smoking CBD-rich joints [68]. On the contrary, another study shows that THC was detectable in blood 4 h after smoking “light cannabis” [42]. In literature, various *T*_max_ and mean peak concentrations (*C*_max_) have been reported, due to the different route and dose of administration. In the studies using low doses of CBD and Δ^9^-THC, the individuals were treated with cannabis decoction receiving 0.3 ± 0.12 mg Δ^9^-THC, and 0.7 ± 0.4 mg CBD per dose, while when treated with cannabis oil they were receiving 1.0 ± 0.2 mg Δ^9^-THC, and 0.9 ± 0.2 mg CBD. In cannabis decoction administration, Δ^9^-THC had a mean *C*_max_ 0.4 ng/mL in serum at a mean *T*_max_ of 1.8 h, while in cannabis oil administration the respective values were 0.4 ng/mL and 1.5 h, respectively. Regarding 11-nor-carboxy-Δ^9^-THC, the mean serum *C*_max_ was 7.4 ng/mL and was found at a mean *T*_max_ of 2.4 h during cannabis decoction administration, while in that of cannabis oil administration the respective values were 5.6 ng/mL and 1.8 h, respectively. Following administration of both herbal preparations 11-nor-carboxy-Δ^9^-THC, but not Δ^9^-THC and CBD, was detected in urine samples of participants at all collection time intervals [61, 62]. After vaporization of CBD-rich cannabis (medical cannabis), *T*_max_ has been reported to be 15 min [68], 0.5 h [42], 0.67 h [57] or 1 h [56], while after the administration of 4 puffs of Sativex, *T*_max_ was achieved in 15 min [65], or 2 h after oral administration of a cannabis extract [58]. Depending on the dose of administration, mean *C*_max_ has been reported to be 6.2 ng/mL when 3.7 mg were received by vaporization [58]. When 1 or 4 cigarettes of light cannabis containing 1.6 and 6.4 mg Δ^9^-THC, respectively, were smoked, *C*_max_ was 9.2 and 15.6 ng/mL [42], while when 6 mg of total Δ^9^-THC (Δ^9^-THC and Δ^9^-tetrahydrocannabinolic acid A) was received by vaporization, *C*_max_ was 24.92 ng/mL [57]. Δ^9^-THC has also been detected after the administration of Epidiolex, a highly purified CBD product, at levels below the LLOQ of 0.125 ng/mL [64], indicating that even trace levels of Δ^9^-THC in a product can lead to detectable levels in blood. Regarding urine, in our study no Δ^9^-THC was detected in urine samples, which was also reported by other authors [68].

In our study, 11-nor-carboxy-Δ^9^-THC was detected in both plasma and urine samples. It was observed that 11-nor-carboxy-Δ^9^-THC was present in the majority of participants’ plasma samples that were collected before administration or 24 h after the previous uptake. Other studies have also reported the presence of 11-nor-carboxy-Δ^9^-THC in blood 24 h after vaporization of medical cannabis [57], or 24 h after the administration of a cannabis extract, that contained 10 mg Δ^9^-THC [29], while in another study of administration of an oil cannabis extract, 11-nor-carboxy-Δ^9^-THC was constantly detected during the 12 h experiment [58]. On the contrary, in a study that participants received medical cannabis by smoking for 10 days, 11-nor-carboxy-Δ^9^-THC was not detectable in blood after 24 h [68]. Regarding urine samples, in our study 11-nor-carboxy-Δ^9^-THC was found in urine samples of all participants collected 3 h post administration, as well as 24 h after administration. The results of our study are consistent with other previous studies that have also reported the presence of 11-nor-carboxy-Δ^9^-THC in urine for at least 24 h. When CBD-rich cannabis that contained 3.7 mg Δ^9^-THC was smoked, 11-nor-carboxy-Δ^9^-THC could be detected in urine up to 5 days, at concentrations ranging between 1.2 and 29.9 ng/mL [56], while in another study of 26 days of smoking CBD-rich cannabis, 11-nor-carboxy-Δ^9^-THC was detectable since the eighth day, after quitting smoking [7]. Positive urine tests for 11-nor-carboxy-Δ^9^-THC have also been observed in patients after receiving FDA-approved formulations. In a 3-month follow-up urine analysis of patients receiving Nabiximols, urine samples showed positive results, despite the low concentrations in blood samples [65]. On the other hand, Pacifici et al. [42] supported that despite the fact that 11-nor-carboxy-Δ^9^-THC was detected 8 h in all participants after smoking light cannabis, it was measurable only in one participant after 24 h in urine, at a concentration of 0.3 ng/mL.

Our results show that CBD probably accumulates in the body and can be detected only in urine, but not in plasma, 24 h after the administration of a CBD formulation. CBD follows rapid metabolism and can be detected in plasma for 2–3 h. This has also been reported by a previous study of administration of CBD-titrated cannabis extracts, where CBD was not detectable in blood 24 h after administration of different CBD formulations [60]. Peak concentrations are reached between 1 and 2 h after administration. Depending on the route and dose of administration, various *T*_max_ have been reported from previous studies. When CBD is administered by vaporization in the form of CBD-rich cannabis or pure CBD, it has been observed that *T*_max_ is reached at 1 h [55], 0.5 h [42], or 0.17 h [57]. After oral intake of CBD, as pure CBD [55], as CBD oil solution or CBD wafers [60], as highly purified CBD (Epidiolex) [64], or nabiximols [60], peak concentrations are reached at 4–5 h post administration, probably due to slower absorption from oral mucosa, compared to smoking. In pediatric patients, *T*_max_ has been reported to be 2 h after oral administration of CBD-rich cannabis extract that is shorter than *T*_max_ reported in adults [59]. Reported peak concentrations of CBD also depend on the dose and route of administration. When 25 mg CBD was administered as sublingual wafers or as nabiximols, *C*_max_ was 9.1 or 4.6 ng/mL, respectively, while when a higher dose of 50 mg administered as a wafer or an oil, *C*_max_ was 15 and 14 ng/mL respectively. When 100 mg CBD was received by vaporization of CBD-rich cannabis or pure CBD or orally as pure CBD, *C*_max_ of 181.4, 104.6 and 11.1 ng/mL were reported [55]. In our study, CBD was detected in all urine samples, for at least 24 h. Other studies support also that CBD can be detected until day 5 after receiving a CBD formulation, pure CBD or CBD-rich cannabis [56].

Overall, the findings of this study are in accordance with findings reported by other previously published studies, although different doses and different routes of administration have been used. This work shows that the repeated uptake of a legal CBD formulation, hemp extract, results to detectable levels of Δ^9^-THC and 11-nor-carboxy-Δ^9^-THC in both plasma and urine. Despite the low levels of Δ^9^-THC contained in the specific formulation, it was likely Δ^9^-THC to be detected in plasma even at low concentrations up to 3 h after the intake of the formulation. This would mean that if an individual was tested for cannabis use between 1 and 3 h after receiving the formulation, it could be accused of being under the influence, since Δ^9^-THC is an active compound. 11-nor-carboxy-Δ^9^-THC was detected in the majority of the participants for 24 h, in both plasma and urine samples, indicating that a positive urine analysis for this compound is possible even 24 h after the CBD product intake.

The results of the present work highlight the possibility of the misinterpretation of a positive result for cannabis in plasma and urine. Despite the low concentrations, Δ^9^-THC is likely to be detected in plasma up to 3 h after the intake of CBD formulations. In the country that shows zero tolerance for the use and the driving under the influence of cannabis, it can be concluded that individuals receiving legal CBD products may be accused of illegal cannabis use.

Although this study has remarkable results, it also has specific limitations. The main limitations are the short interval of the study design and the number of participants. The present work shows the effect of the daily uptake of a CBD formulation before and the next 3 h after the daily administration. Moreover, a comparative work with cannabis users would be helpful, to draw conclusions if the differentiation between legal and illegal use of cannabis products is feasible. Further work is required to determine indicators for the differentiation of legal and illegal use of cannabis products.

## Conclusions

The results showed that Δ^9^-THC is likely to be found in plasma although at low concentrations. The detection of 11-nor-carboxy-Δ^9^-THC in plasma samples obtained before the last intake of CBD oil in 8 out of 10 patients verifies its accumulation in the body. In addition, the detection of 11-nor-carboxy-Δ^9^-THC in both urine and plasma of the subjects of this study raises questions and concerns for the proper interpretation of toxicological results, especially considering Greece’s zero tolerance law applied in DUID and workplace cases. For this reason, it is necessary to determine new biomarkers during the investigation of forensic cases to differentiate patients under CBD treatment from illegal users of cannabis.
